# Process-outcome relations in music therapy versus music listening for people with schizophrenia viewed through a mediational model: the role of the therapeutic alliance

**DOI:** 10.3389/fpsyt.2023.1120003

**Published:** 2023-05-02

**Authors:** Niels Hannibal, Inge Nygaard Pedersen, Lars Rye Bertelsen, René Ernst Nielsen, Christian Gold

**Affiliations:** ^1^Department of Communication and Psychology, Aalborg University, Aalborg, Denmark; ^2^Aalborg University Hospital - Psychiatry, Aalborg, Denmark; ^3^The Music Therapy Research Clinic, Aalborg University, Aalborg, Denmark; ^4^Department of Clinical Medicine, Aalborg University, Aalborg, Denmark; ^5^Norwegian Research Centre (NORCE), Bergen, Norway; ^6^Department of Clinical and Health Psychology, University of Vienna, Vienna, Austria

**Keywords:** schizophrenia, attendance, drop out, negative symptoms, music therapy, music listening, assessor- and patient- blinded study

## Abstract

**Objectives:**

Examine whether change in clinical outcomes for patients with schizophrenia and negative symptoms randomized to either Music Therapy (MT) or Music Listening (ML) is associated to moderators and mediators, with focus on alliance, attendance and dropout.

**Method:**

An exploratory post-hoc analysis of data from an original randomized controlled trial (RCT) investigating the effect of MT vs. ML for people with schizophrenia and negative symptoms. Inclusion to the study was implemented through screening of referred patients for symptoms of schizophrenia and negative symptoms. A total of 57 patients were randomly assigned, 28 to MT and 29 to ML. Session logs and notes were included in this study. Statistical analysis investigated moderator and mediator relation to outcome variables: Negative symptoms, functioning, quality of life, and retention to treatment.

**Results:**

On average, participants in MT attended 18.86 sessions (SD = 7.17), whereas those in ML attended 12.26 (SD = 9.52), a statistically significant difference (*p* = 0.0078). Dropout at 25 weeks was predicted by intervention, with dropout being 2.65 (SE = 1.01) times more likely in ML than in music therapy (*p* = 0.009). Helping alliance score at weeks was explained by intervention, with mean score being 0.68 (SE = 0.32) points lower in ML than in MT (*p* = 0.042). The number of sessions attended was also explained by intervention, with participants in ML attending on average 6.17 (SE = 2.24) fewer sessions than those randomized to MT (*p* = 0.008). Though both groups improved significantly, improvements in negative symptoms, depression, and functioning tended to be higher in ML, whereas improvements in alliance and quality of life tended to be higher in MT.

**Conclusion:**

The analysis could not detect a direct link between helping alliance score and outcome variables. However, the analysis documented a stronger alliance developed in the MT group, a lower dropout rate, as well as higher attendance in treatment.

**Clinical Trial Registration**: www.ClinicalTrials.gov, identifier: NCT02942459.

## 1. Introduction

Developing an alliance in therapy with individuals diagnosed with schizophrenia is important. The concept of the therapeutic alliance used in this study is based on the definition made by Bordin (1). The alliance is understood as a common factor to psychotherapy and also to music therapy (2) where the relationship between patient and therapist is viewed in the perspective of shared understanding of therapeutic goals, tasks and bonds. More present literature (3) describes the importance of the alliance: “A strong alliance indicates that the patient accepts the treatment and is working together with the therapist, creating confidence in the patient that the treatment will be successful [(3), p. 271]. As far back as 2006 Coture (4) stated that for individuals with schizophrenia, “the therapeutic alliance is related to global functioning, reduced symptom severity, a better quality of life, improved social functioning, and greater medication compliance” [(1), p. 10]. Witthof (5) concluded in their study that emotional and collaborative relationship is essential for a positive outcome in psychotherapy. They also found that negative symptoms which seem similar to a detached style of interaction, were found to be related to a poorer alliance in non-psychotic samples. The level of negative symptoms in individuals with schizophrenia seems a major factor in a successful psychotherapy treatment within this population. Shattock (6) found in a systematic review “for therapist and client-rated [alliance] predicting overall symptomatic outcomes” [(6), p. e80]. They stated that “Establishing good quality alliance may prevent disengagement from services, which is a key issue for people with psychosis” [(6), p. e81]. Jung (7) found in a study with 56 patients “negative symptoms to be a relevant predictor for patient and therapist rated alliance” [(7). p. 177] and proposed that negative symptoms would be a barrier for the development of a therapeutic alliance.

Based on this developing a therapeutic alliance with a target population specifically recruited because of negative symptoms would be difficult and the quality of the early alliance would predict the outcome of the treatment.

A recent randomized controlled trial (RCT) investigated the effect of music therapy (MT) in this population (8). It was conducted in psychiatric settings in Denmark and had a unique design where both intervention and screening were blinded and an active control group offering music listening (ML) was included. The study showed no effects between groups on the primary outcome of negative symptoms [Positive and Negative Symptoms Scale (PANSS) negative subscale], with similar improvements in both groups. There were also no between-group differences on secondary outcomes [Brief Negative Symptom Scale (BNSS) and Global Assessment of Functioning (GAF)]. There were some differences in the Quality-of-Life scale (WHOQOL-Brief) for the group receiving music therapy intervention. Finally, the helping alliance as measured using Helping Alliance Questionnaire (Haq-II) showed a tendency toward a between-group difference. Those allocated to MT had a Haq-II score of 5.0 (95% CI 4.6–5.3) after 5 sessions, compared to a somewhat lower score of 4.7 (95% CI 4.4–5.1) in ML. The average Haq-II score of 5.0 indicates that those in MT had developed a sufficient alliance after 5 sessions a value of >4.92 Haq-II score was suggested by (9, 10), whereas those in ML had not, albeit with no statistically significant difference between the groups. The MT group continued to improve the alliance over all 25 sessions, whereas the ML’s alliance first improved until the 15th session but was lower after 25 sessions. The control group also never reached the threshold for a strong alliance score. As might be expected in this population, dropout rates were high in both groups, but tended to be higher in the ML group compared to the MT group. At 25 sessions, 39% (11/28) of the MT group and 55% (16/29) in the ML group had either dropped out or left the study for other reasons. The dropout rate was higher in this material than in smaller-scale descriptive studies (11–14), comparatively low drop-out in MT for people with schizophrenia both in hospital and social psychiatry. In these studies, the attendance to treatment was relatively high (86–90%), but here no data on how the alliance developed was available. Including both dropout and attendance in the analysis of the population in this study was found warrant for further investigation of process-outcome relations. It is the first study where data on the therapeutic alliance, the level of attendance and outcome are combined, and it offers a unique opportunity to investigate if attendance is related to the development of an alliance between patient and therapist in music therapy.

### 1.1. Aims and hypotheses

The aim of this study was to examine whether helping alliance mediates changes in clinical outcomes in MT vs. ML. Do age, duration of illness, and gender influence the outcome of the study, and how does the working alliance measured at 5th, 15th, 25th session, the number of attended sessions, and dropout from treatment influence the outcomes negative symptoms, quality of life, functioning, and retention in treatment?

**Figure 1:**
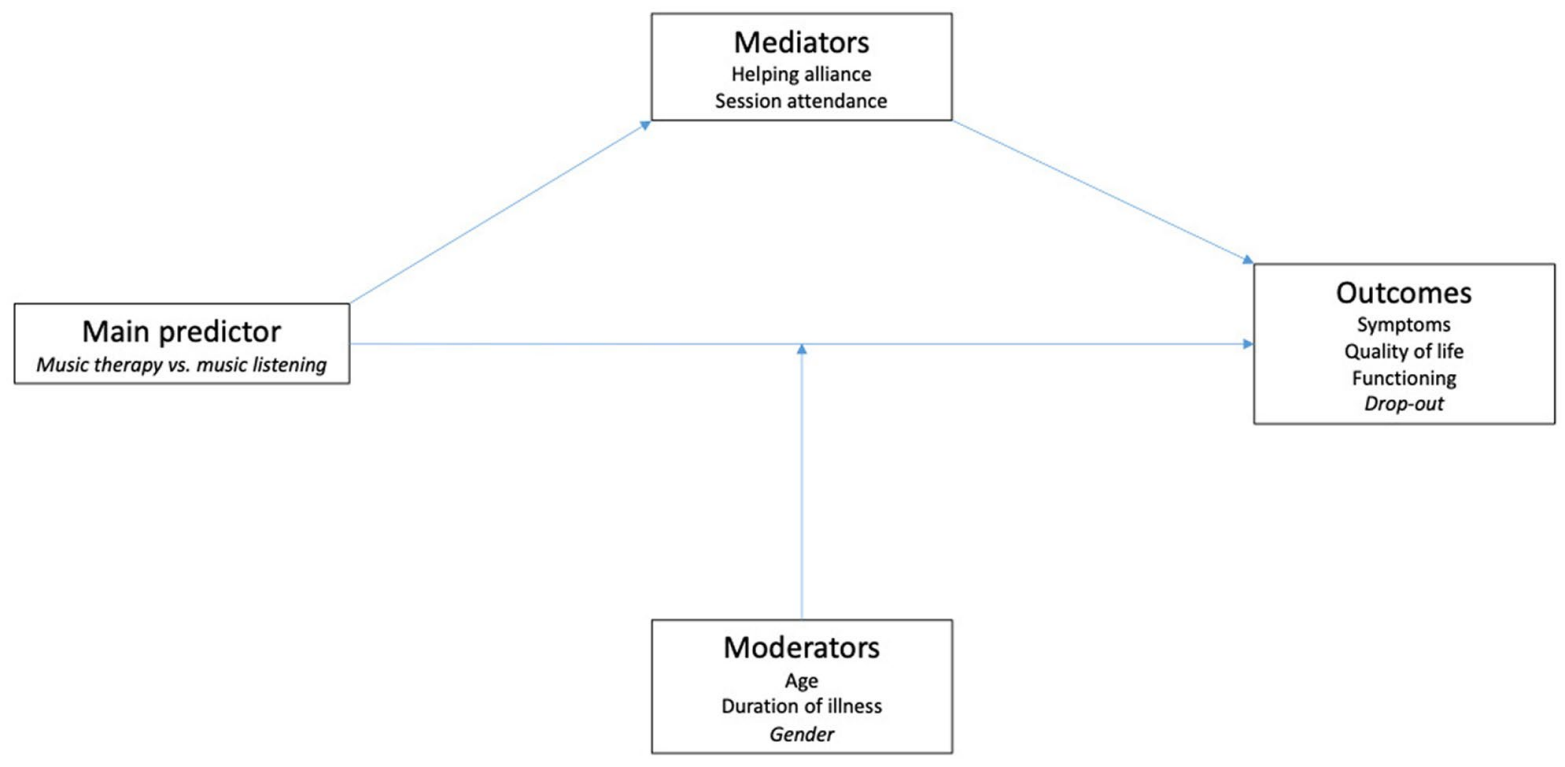
Conceptual mediational model of possible process-outcome relations in music interventions for people with schizophrenia. Continuous variables are shown in plain font, binary variables in italics. Helping alliance was measured at several time points (5, 15, 25 sessions).

Specifically, we aimed to examine the relation between (1) the intervention to which participants were randomized (MT vs. ML; main predictor) (2); age (in years), duration of illness (in years), gender (male/female; moderators) (3); working alliance (Haq-II) measured at sessions 5, 15, and 25; number of sessions attended/canceled (mediators); and (4) symptoms, functioning (continuous outcomes, measured as endpoints), quality of life, and retention in treatment (binary outcome). The hypothesized relations between predictor, moderators, mediators, and outcomes are shown in Figure 1. We expected MT to be associated with higher helping alliance than ML, and higher helping alliance in turn to be associated with lower symptoms, higher quality of life, higher level of functioning, and higher retention in treatment (i.e., lower risk of dropout). Regarding the moderators, we did not have a directional hypothesis regarding age, duration of illness, or gender.

## 2. Method and materials

The present study is based on an exploratory post-hoc analysis of data from the original RCT already described in detail (8, 15). The study by Pedersen et al. was a parallel RCT design with two interventions: Individual music therapy (active and receptive methods) with a trained music therapist and music listening (only receptive) with an experienced social worker (care person). Inclusion to the study was through screening of referred patients for symptoms of schizophrenia and negative symptoms. A total of 57 patients were randomly assigned, 28 to music therapy and 29 to music listening. A total of 29 participants completed the study and were followed-up at the final 25-week time point, divided in to 17 in the music therapy group and 12 in the music listening group.

### 2.1. Design

A randomized controlled trial design with an experimental group (music therapy with a music therapist) and an active control group (music listening with trained care person).

### 2.2. Participants

All referred participants were screened for negative symptoms using PANSS (described below) by a blinded screener. Patients were also screened for depression and would be excluded if depression was found to avoid negative symptoms being in relation to depression. Other exclusion criteria were if the patient was first diagnosed with schizophrenia <2 years ago and had experienced hospitalization within the last tree month. The population in this study consisted of 59 participants, randomly assigned to either music therapy or music listening condition (For description of the interventions see below and Supplementary material).

All participants were informed that they would receive a music therapy activity as a way of blinding them. Participants were excluded if there was more than 30 days between sessions or if they failed to attend more than five sessions. Participants were recruited from the Region of North Jutland and Region of Copenhagen.

### 2.3. Interventions

All participants were randomly assigned to 25 weekly sessions of either individual music therapy (MT) or individual music listening (ML) being together with care person, where it was possible to listen to specific playlists of music developed by music therapists. MT was conducted by six experienced music therapists who all had a five-year master’s degree in music therapy from Aalborg University. This music therapy program has a psychodynamic profile, where developing the therapeutic relationship through musical means (active and receptive) is an essential part of the training. The music therapists had graduated between 1997 and 2016. All music therapy providers had minimum 2 years of clinical experience with this population.

ML was conducted by seven care persons, who had no formal music therapy training but were familiar with persons with schizophrenia. Four of them were professionally trained social workers and three had personal or clinical experience with this population. To maintain blinding of participants, those conducting both interventions were referred to as “therapists” and both interventions were introduced to the participants as “music therapy activities” (listening to music playlists was one possible activity in both interventions).

Both interventions were manualized, and the providers were trained in how to perform the interventions. All providers received in all 13 h of prior training. A summary of the manuals used for both interventions in this study is provided in Supplementary material (Appendix I). The full manuals are developed by the principal investigator (co-author INP). Both manuals contained guiding principles for the providers as suggested for complex interventions (16). The manual for MT was structured in four levels according to the recommendation by Waltz, et al. (16). A similar structure has been used in other music therapy manuals (17–19). The four levels were: unique and essential; essential but not unique; acceptable but not necessary; and not acceptable–proscribed therapeutic principles. The music therapy sessions took place in a room with instruments (e.g., piano, guitar, percussion, song books). In this study the music therapists were focused on relation and alliance building through engaging and motivating the patient to participate through active and receptive interventions. Active musical activities to engage with the patient such as improvisation, song writing and performing constitutes a music therapy session. This therapeutic process unfolds as a collaboration between therapist and patient, where the therapist facilitates the use of instruments. Active music playing and improvising activates the patient. The sound and playing form a matrix for the relationship dynamics. In this context communication unfolds without the need for language, and meaning is also related to experience and esthetics. In song writing and song performing words serves as poetic and symbolic representations. Receptive music interventions were used to regulate arousal, sharing experience and stimulate mental activity and consisted of playlist from the Music Star app and Spotify when participants own music were used. The Music Star app contain music with minimal to medium stimulation and all the music available is considered as supportive music according to the taxonomy for music therapy and imagery (20).

The manual for ML had only three levels. What must be done, what could be done, and what was not allowed. It was not considered necessary to distinguish level one and two in the control condition because there were no unique elements. The only music available was through the Music Star app.

One thing was common for both manuals: If needed therapist should enforce the rule of participants not being intoxicated and not smoking during sessions, and make sure that the mobile phone did not disturb. In order to improve the blinding of the intervention a repertoire of fixed replies to questions from the patients was also prepared. The manual for MT was focused on the therapist attitude and focus of attention of the therapist during treatment: Disciplined subjective, use of the relation and timing. Beside this the music therapist should engage and motivate the participants. Using the relationship was related to using music in any way possible to engage and interact with the patients. The manual for ML was developed to match the MT intervention, but without any specific therapeutic agenda. ML was conducted as passive and non-inquisitive as possible, and the purpose was to monitor the effect on negative symptoms in an environment where any initiative and activity would originate from the patient. Basically, ML offered companionship and entertaining activities.

### 2.4. Moderators

Patients age, the duration of their illness and gender were included as moderators in the analysis.

### 2.5. Mediators

#### 2.5.1. Working alliance (Haq-II) measured at sessions 5, 15, and 25

Working alliance was an outcome variable in the original study but is included as a mediator in this analysis. The number of sessions attended was also used as a mediator. The Helping Alliance Questionnaire-II (Haq-II) was originally developed by Luborsky et al. (21). Its 19 items inquire about the participant’s experience of collaboration, perceptions about the therapist, motivation, and the participant’s feelings about the therapist. This is in accordance with theory about the therapeutic alliance (22–24). Each item is scored on a Likert-type scale format (strongly disagree, disagree, slightly disagree, slightly agree, agree, and strongly agree) which is converted to a scale from 1 to 6. To compute the overall Haq-II score, five items (items 4, 9, 11, 16, and 19) must be reversed. The sum score is divided by the number of items (i.e., 19) to produce the final Haq-II score. The Danish version of the Haq-II scale for patients’ perspectives was developed through back-to-back translation, and its feasibility was tested on a music therapy population of patients with both non-psychotic and psychotic conditions (2). Furthermore, it was not found relevant to include the version of the therapists’ perspectives as the focus in the original study was to only monitor the patient perspective of the relation through the alliance score. Therefore no effort was made to produce a valid translation of Haq-II for therapists. A therapeutic Haq-II therapist perspectives could in a future study be relevant if there is more focus on agreement between therapist and patient, and focus on investigating any relation between therapeutic interventions, method and forming of the alliance over a treatment trajectory. This analysis showed acceptable internal consistency. The Haq-II questionnaire is according to Paap et al. (24) comparable to the Working Alliance Inventory (WAI) which is one of most used alliance assessment instruments (25). The Haq-II has been used in the study of dropout and the therapeutic alliance by Johansson & Eklund (9). In a study with a population of mixed diagnoses (*n* = 166), the mean Haq-II score for completers was 4.92, compared to 4.58 for patients who droppedout early from treatment [(9), p. 640]. We used this value as a benchmark for adequate alliance formed during treatment in this study.

#### 2.5.2. Number of sessions attended/canceled

The number of sessions attended was analyzed as reported by intervention providers. In a few cases where the exact number of sessions attended could not be reconstructed (3 participants in ML), we assumed that no sessions had been attended when the participants withdrew consent shortly after randomization (2 participants). In another case, the participant stopped after approximately 5 sessions and we used that as the most likely number.

### 2.6. Outcomes used in mediational model

#### 2.6.1. Symptoms

The outcome variables used in this study are described in detail in Pedersen et al. (8). Briefly, the Positive and Negative Syndrome Scale (PANSS) is a scale consisting of seven items for positive symptoms, seven items for negative symptoms and for 16 items for the evaluation of general psychopathology. The PANSS scale is validated and verified and is standard in many outcome studies (26–28). In this study, evaluation was based on a structured clinical interview (SCI-PANSS), which is designed to help the interviewer in getting relevant information needed to complete the rating. Interrater reliability is generally high on the full PANSS scale score. Intraclass coefficients was 0.98–0.99, and the interclass coefficient is acceptable between 0.83–0.90 (29). High scores on the PANSS indicate high symptom severity.

The Brief Negative Symptom Scale (BNSS) consists of 13 items that pertain to 6 subscales on anhedonia, distress, antisociality, avolition, blunted affect, and alogia. This scale differs from PANSS as it separates appetitive and consummatory anhedonia, antisociality and internal experience. Its aim is a threefold examination of the knowledge of behavior, of the social context, and of the report of the experience of the participant concerning everyday life (30). High scores on the BNSS indicate higher symptom severity.

#### 2.6.2. Functioning

The Global Assessment of Functioning (GAF) scale is a standard method for clinical evaluation of the participant’s overall level of function. It includes psychological, social, interpersonal, and occupational functioning in regard to a patient’s mental-health condition. The scale ranges from 0 to 100. High score equals high function (31).

#### 2.6.3. Quality of life

WHO-Quality of Life brief scale (WHOQOL-Brief) is a self-reported questionnaire containing 26 items rated on a Likert-type scale ranging from zero to five. Zero is very poor and five is very good. The analysis produces a total score, and a score for the physical, psychological, social, and environmental domain (32).

#### 2.6.4. Retention in treatment

All session logs and notes were revisited, and every session registered to identify level of attendance to treatment, when treatment was terminated and if possible, identifying the reason for termination. In the RCT protocol termination of treatment could be registered in five ways: Completer, wish to stop, non-compliance, drug/substance abuse and other. “Other” was defined as situations where the termination from treatment was grounded in conditions not influenced by the participant, such as moving, change in medication, COVID-19 and hospitalization due to severity in health condition. We also looked at any course of continuation for each group.

### 2.7. Other outcomes (not used in mediational model)

The Calgary Depression Scale for Schizophrenia (CDSS) was included in the original study to exclude patients who suffered from depression and negative symptoms as depression symptoms are difficult to differentiate from the negative symptoms of schizophrenia. The CDSS consists of nine items. Each item can be given a score between 0 and 3, and a total score between 0 and 27. Light depression is defined as a score between 3 and 6. Moderate depression is defined as a score between 7 and 10, and severe depression is defined as a total score over 10. The CDSS is reliable and valid for the evaluation of depression in schizophrenia, and in differentiating depression from negative symptoms when combined with PANSS (33).

### 2.8. Statistical analyzes

Descriptive analysis used means (SDs) for continuous variables (e.g., sessions attended, helping alliance) and counts (percentages) for binary variables (e.g., retention in treatment versus dropout and reasons for dropout). Changes over time within groups were analyzed as means (95% CI) and transformed into effect sizes for interpretation. Inferential statistics used a two-sided 5% significance level. No multiplicity adjustment was used, as all analyzes were exploratory. Effect sizes were interpreted as small (0.20), medium (0.50), and large (0.80). To explore possible relations between the interventions, moderators, mediators, and outcomes (Figure 1), we used linear models for predictors of continuous outcomes, and generalized linear models (binomial) for predictors of the binary outcome (dropout).

## 3. Results

The baseline characteristics was a total of 57 participants (34 male) were randomized to MT (*n* = 28) or ML (*n* = 29). Baseline characteristics were similar between the groups, as described in Pedersen et al. (8). Briefly, mean age was 41 years (SD = 13) in MT and 37 years (SD = 11) in ML; mean duration of illness was 9 years (SD = 8) in MT and 7 years (SD = 9) in ML; 13 participants (23%) had completed law-mandated school, 18 (32%) grammar school. The most common diagnostic subtype was paranoid schizophrenia. GAF scores around 40 at baseline indicated that the participants were severely challenged in their daily functioning.

### 3.1. Session attendance and dropout in MT versus ML

**Figure 2:**
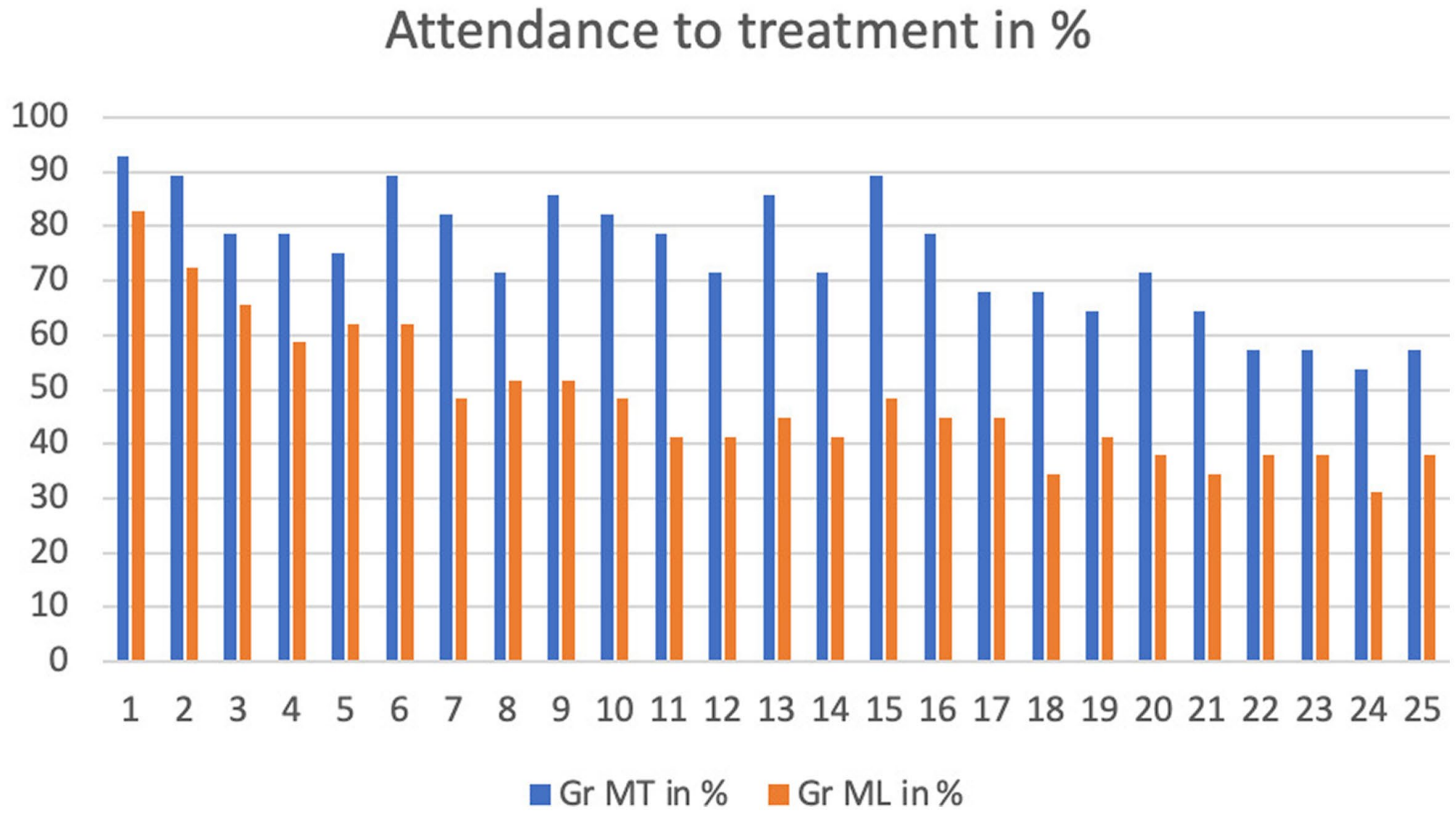
Attendance to treatment for each session in %.

On average, participants in MT attended 18.86 sessions (SD = 7.17), whereas those in ML attended 12.26 (SD = 9.52), a statistically significant difference (*p* = 0.0078). Figure 2 illustrates the percentage of possible sessions attended in each group. From session 1 to session 15 between 90 to 70% of session in MT executed; from session 16 to 25 this number decreased to between 70 to 55%. In ML, around or under 50% of sessions were executed from session 7 onwards.

**Figure 3:**
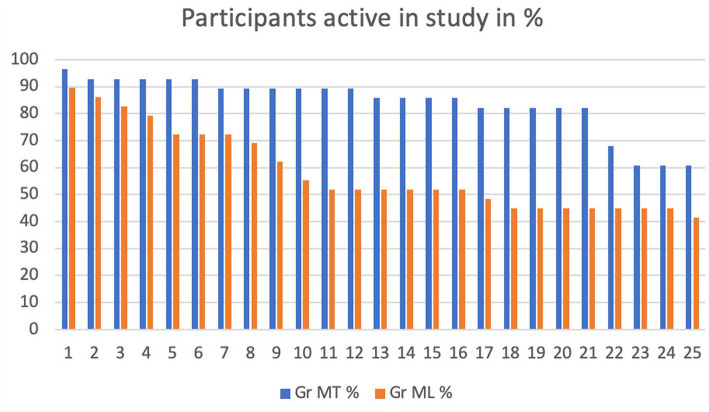
Participants active in study in %.

Figure 3 illustrates the difference in discontinuation for any reason in both groups. In the MT group over 80% participated up to session 21. In the last four sessions the group is decreased to 64%. In the ML group 25% had left the study after 5 sessions and only 41% completed.

A comparison of dropout rates with reasons for dropout (Table 1) indicates that eight (28%) participants expressed a wish to terminate ML, compared to only one (4%) in MT. Rates of non-compliance or termination for other reasons were similar between interventions.

**Table 1:** Completion of treatment.

Intervention group	Completed treatment	Participant’s wish to terminate	Non-compliant	Other	Total
Music therapy	17 (61%)	1 (4%)	4 (14%)	6 (21%)	28
Music listening	12 (41%)	8 (28%)	4 (14%)	5 (17%)	29

“Other” was defined as situations where the reason for termination of treatment was grounded in conditions not actively influenced by the participant (such as moving, change in medication, COVID-19, and hospitalization due to severity in health condition).

### 3.2. Helping alliance in MT versus ML

**Figure 4:**
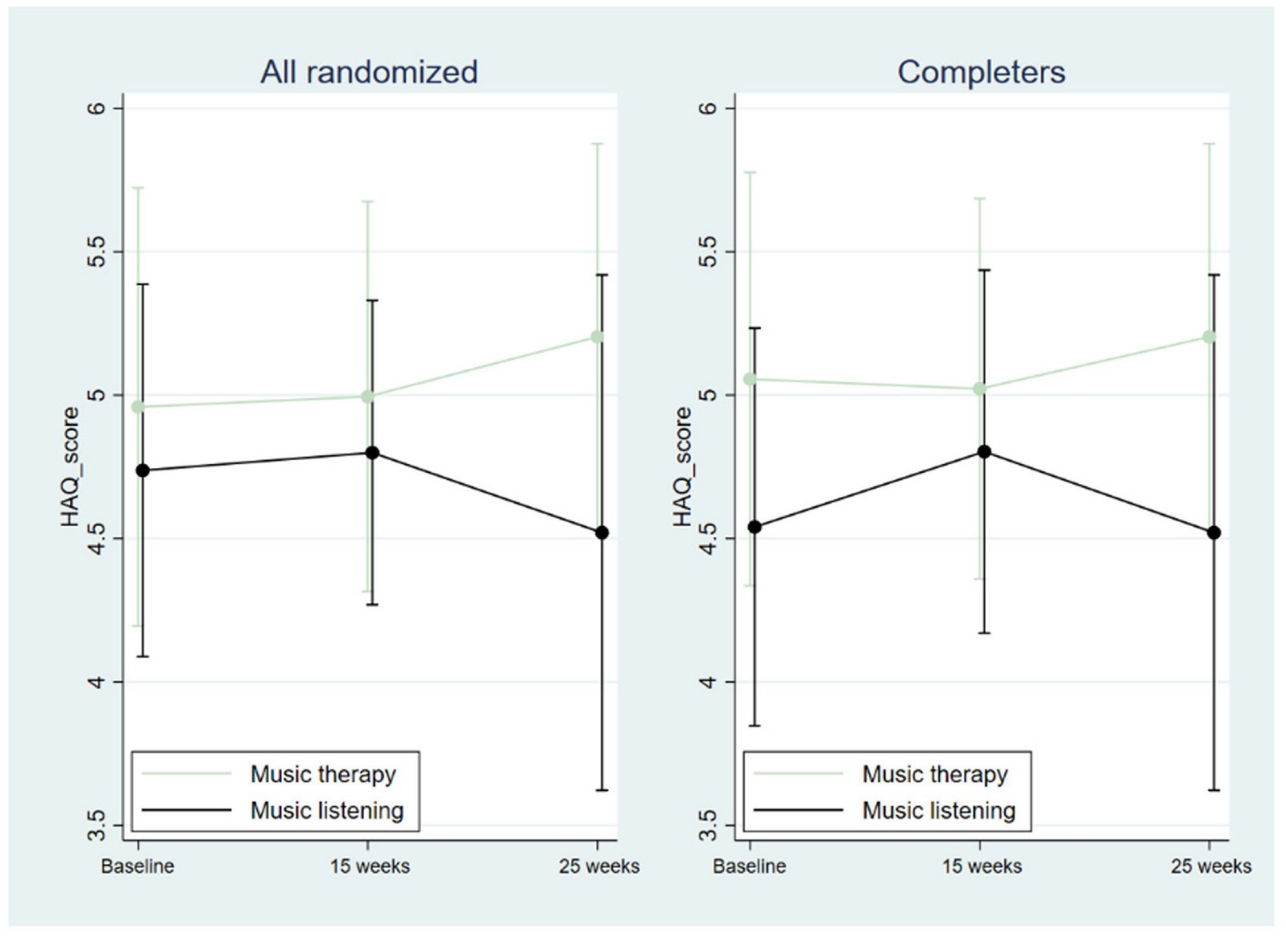
Helping alliance (Haq-II) scores. Vertical lines indicate standard deviations.

Descriptive analyzes of Haq-II scores (Table 2, Figure 4) showed that alliance tended to be higher in MT than in ML at all time points. The mean alliance score in MT reached an adequate value (> 4.92) after 5 sessions (6, 7).

**Table 2:** Helping alliance (Haq-II) scores in each intervention.

Session	Music therapy		Music listening		value of *p*	*n*	*M* (SD)	*n*	M (SD)
5	24	4.96 (0.76)	19	4.74 (0.65)	0.31
15	23	4.99 (0.68)	12	4.80 (0.53)	0.36
25	16	5.20 (0.67)	9	4.52 (0.90)	0.07

### 3.3. Changes over time between groups and within groups

As reported previously, between-group differences were non-significant for the primary outcome PANSS negative subscale, mean difference − 0.24, 95% CI −1.76 to 1.27, *p* = 0.754 in the intention-to-treat analysis: −0.98, 95% CI −5.06 to 3.09, *p* = 0.625 when only analyzing completers (8). When examining within-group change over time, improvements were found in both interventions. Effect sizes were in the large range in PANSS total and negative subscale; changes in positive and general symptoms were smaller (Table 3). Improvements in BNSS, depression, and functioning tended to be higher in ML, whereas improvements in quality of life tended to be higher in MT (Table 3).

**Table 3:** Within-group analysis of changes over time in each intervention.

Variable	Original scale		Effect size (Glass’ ∆)		Music therapy M [95% CI], *p*	Music listening M [95% CI], *p*	Music therapy	Music listening
PANSS total	−9.8 [−17.7, −1.9], 0.019*	−9.5 [−14.5, −4.5], 0.001**	−0.87	−0.85
PANSS negative subscale	−4.2 [−7.2, −1.2], 0.009**	−5.5 [−8.2, −2.9], 0.001***	−0.88	−1.15
PANSS positive	−1.8 [−3.7, 0.2], 0.074	−0.3 [−1.9, 1.3], 0.684	−0.38	−0.07
PANSS general	−3.8 [−8.2, 0.7], 0.093	−3.7 [−6.9, −0.5], 0.028*	−0.52	−0.51
BNSS total	−3.9 [−8.8, 0.9], 0.106	−7.5 [−15, −0.1], 0.047*	−0.41	−0.79
BNSS anhedonia subscale	−2.1 [−3.8, −0.5], 0.015*	−2.9 [−4.8, −1], 0.006**	−0.75	−1.03
BNSS distress subscale	−0.6 [−1.6, 0.4], 0.243	0 [−1.2, 1.2], 1	−0.37	0
BNSS asociality subscale	−0.8 [−1.7, 0.2], 0.103	−1.2 [−2.1, −0.2], 0.019*	−0.38	−0.58
BNSS avolition subscale	−0.9 [−2.1, 0.3], 0.14	−1.5 [−2.7, −0.3], 0.019*	−0.5	−0.85
BNSS blunted affect subscale	0 [−1.2, 1.2], 1	−2.1 [−5, 0.8], 0.139	0	−0.57
BNSS alogia subscale	0.4 [−1.4, 2.2], 0.639	−0.5 [−1.8, 0.9], 0.461	0.15	−0.17
Calgary Depression Scale for Schizophrenia	1.1 [−1.4, 3.6], 0.377	1.7 [−0.6, 4], 0.134	0.48	0.75
GAF	2.8 [−1.6, 7.2], 0.202	5.2 [−1.9, 12.2], 0.135	0.38	0.72
WHO-QOL, total score	7.5 [0.2, 14.8], 0.044*	2.8 [−2.8, 8.5], 0.294	0.59	0.22
WHO-QOL physical health domain, raw score	3.1 [0.3, 5.8], 0.03*	0.3 [−1.3, 1.9], 0.653	0.61	0.07
WHO-QOL psychological domain, raw score	2.5 [0.4, 4.7], 0.025*	1.2 [−1.2, 3.5], 0.3	0.58	0.27
WHO-QOL social relationships domain, raw score	0.5 [−0.7, 1.8], 0.391	−0.6 [−2.1, 0.8], 0.341	0.27	−0.32
WHO-QOL environment domain, raw score	1.8 [−0.1, 3.6], 0.059	0.4 [−1.7, 2.5], 0.672	0.41	0.1
Haq-II, Helping Alliance Questionnaire	0.1 [−0.1, 0.4], 0.271	0 [−0.6, 0.5], 0.937	0.21	−0.03

Effect sizes were calculated by dividing the mean change by the baseline SD of the whole sample (Glass’ ∆). *p < 0.05, **p < 0.01, ***p < 0.001.

### 3.4. Mediation analysis: linear and generalized linear models

Significant relations between intervention, moderators, mediators, and outcomes are shown in Table 4; a full list of all relations examined is found in Appendix II. The first group of models tested whether outcomes were explained by intervention and moderators. Dropout at 25 weeks was predicted by intervention, with dropout being 2.65 (SE = 1.01) times more likely in ML group than in MT group (*p* = 0.009; Table 4). No significant effects were found in the models including an interaction between intervention and moderators, suggesting that the intervention effect did not depend on age, disease length, or gender (Table 4; Appendix II).

**Table 4:** Linear and generalized linear models (selection).

Model/predictor	Estimate (SE)	*p*-value
1. (a) Outcome explained by intervention and moderators		
Dropout before 25 weeks		
Group	2.65 (1.01)	0.009**
Age	−0.01 (0.06)	0.803
Duration of illness	0.05 (0.09)	0.601
Gender	1.36 (0.99)	0.169
2. (a) Mediator explained by intervention		
Alliance (week 25)		
Group	−0.68 (0.32)	0.042*
Sessions		
Group	−6.17 (2.24)	0.008**
2. (b) Mediator explained by intervention x moderators		
Sessions		
Group	−35.92 (16.42)	0.039*
Age	−0.77 (0.48)	0.119
Duration of illness	0.82 (0.63)	0.206
Gender	−0.99 (5.63)	0.862
Group x age	0.96 (0.54)	0.087
Group x duration of illness	−0.6 (0.7)	0.401
Group x gender	−2.47 (7.29)	0.737
(3) Outcome explained by mediators		
Dropout before 25 weeks^1^		
Alliance (week 5)	−0.67 (1.13)	0.556
Alliance (week 15)	1.4 (1.4)	0.318
Sessions	−0.45 (0.19)	0.016*

Showing results of linear models (for continuous outcomes) and generalized linear models (for binary outcomes). Model intercepts not shown. Reference categories: gender: female; group: MT. Age and duration were measured in years. ^1^Not possible for alliance at week 25. This table shows only models yielding at least one significant results; all models are shown in Appendix II.

The second group of models tested whether mediators were explained by intervention. Alliance (Haq-II score) at 25 weeks was explained by intervention, with mean Haq-II score being 0.68 (SE = 0.32) points lower in ML than in MT (*p* = 0.042; Table 4). The number of sessions attended was also explained by intervention, with participants in ML attending on average 6.17 (SE = 2.24) fewer sessions than those randomized to MT (*p* = 0.008; Table 4). This effect was also present in the full model with all interaction effects (Table 4); however, since none of these interaction effects were significant, we relied on the simpler model for interpretation.

Finally, the third group of models tested whether outcomes were explained by mediators. Although any potential effects of alliance on outcomes did not become significant, the number of sessions attended was related to the likelihood of dropout (estimate −0.45, SE = 0.19, *p* = 0.016; Table 4), indicating that those dropping out before 25 weeks had attended a lower number of sessions than those who did not.

**Figure 5:**
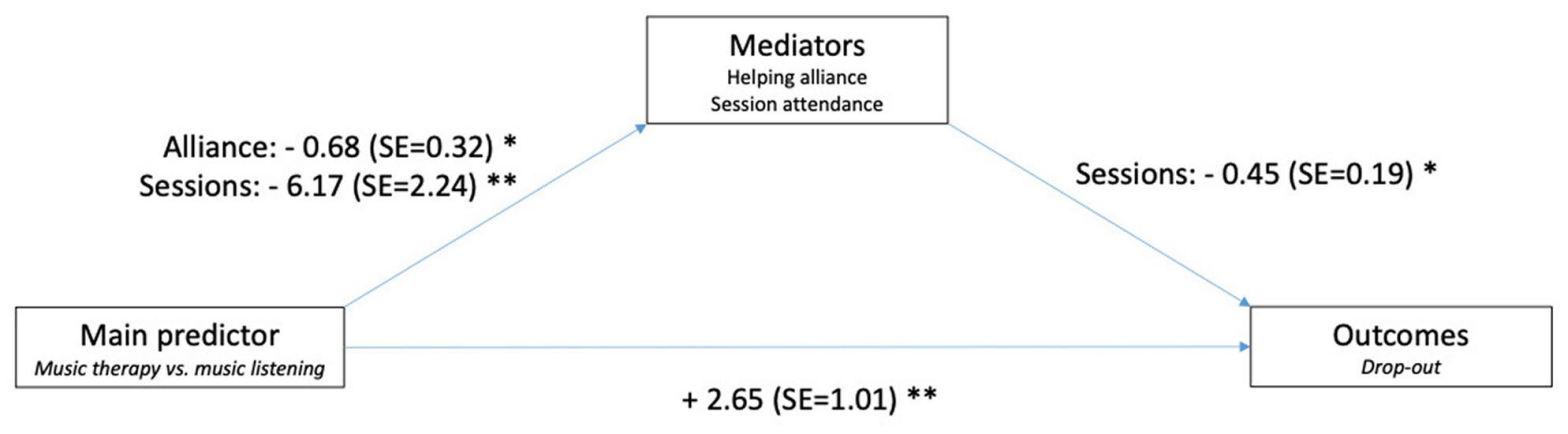
Illustration of final mediational model.

These findings are illustrated graphically in Figure 5, which represents the final mediational model. Non-significant variables from the initial model are omitted here. To summarize, ML was associated with higher dropout than MT, and this was partly explained by differences in helping alliance and number of sessions attended. ML was associated with lower alliance and fewer sessions than MT, and fewer sessions were in turn associated with higher risk of dropout (Figure 5).

## 4. Discussion

In this study of the role of helping alliance in music therapy versus music listening for people with schizophrenia, we found some evidence to suggest that MT was associated with better helping alliance, higher attendance, and higher retention in treatment (i.e., lower dropout) than ML. As illustrated in Figure 5, we found a significant positive relation between the intervention type as the main predictor and both mediators (helping alliance, session attendance), and between the main predictor and one of the outcomes (retention in treatment). Haq-II scores at 25 sessions were 0.68 points higher in those randomized to MT than in ML. Additionally, those randomized to MT attended on average 6 sessions more than those randomized to ML. Finally, those randomized to MT were more likely to complete treatment than those in ML.

Other relations postulated in our initial model (Figure 1) could not be confirmed: We did not find significant moderating effects of age, duration of illness, or gender. This might mean that the results apply across the broad sample. We also did not find a significant relation between mediators and other outcomes. However, as the importance of helping alliance for clinical outcomes is a known and well-replicated finding from other studies (3), we think that the most likely explanation for this null finding is limited power. The sample size was less than originally planned and generally too low to reliably detect important effects.

Comparison between MT and an active control for schizophrenia is not commonly reported in the literature (34, 35). There are a few studies where the intervention is conducted by a care person (a nurse or another person without a formal MT training), but they are all with inpatients and in group format (35). In this perspective the findings from the ML intervention are noteworthy. Despite of a substantial dropout rate, with only 42% completing all 25 sessions in the ML group, a significant within-group reduction in PANSS negative subscale was observed. Knowing that the non-music therapist who conducted this intervention were instructed to be as non-directive as possible, this apparently had a positive effect on some of the participants. This underlines the importance of offering this kind of intervention. But the ML group did not develop a strong alliance to their therapist. Actually 27% of the participants that dropped out from the ML group did it before the 10 sessions. Why this happened is not known as there are only reported a wish to stop but not the reason why. The findings are ambiguous and do not give indications that could predict who would benefit from music therapy and who would benefit from music listening. However, it is an important finding that positive change in negative symptoms for this population is possible even though a strong alliance is not found.

When looking at the MT intervention the findings support the intention in the manual to continuously motivate the participant to engage and participate in musical activities without using pressure or persuasion viewed as an increase of the alliance between patient and therapist, in the much higher amount of completed sessions, and in the lower dropout from the MT intervention. These guidelines reflect over 20 years of clinical experience with this population and were developed in a collaboration with 15 Danish music therapists having this experience. They show a combination of a psychodynamic inspired understanding of internal dynamics, the importance of the therapist’s awareness of his/her attitude toward the patient, and an emphasis on using the musical means as a way of engaging, activating, interacting, communicating and relationship building with the patient. There are traces of mentalization based treatment and process-oriented music therapy (18).

Both Couture (4) and Witthorf (5) state that the alliance is related to function and outcome of treatment. The findings in this study are ambiguous as both groups improved their symptoms and only one group developed a strong alliance. The findings correspond with the literature.

Shattock (6) confirming that low alliance is associated with disengagement from treatment as observed in the ML group. However, this material also documents that improvement is possible without a strong alliance as seen in the same group. The findings document that the music therapy has high attendance, a growing alliance, lower level of negative symptoms after treatment and higher quality of life. This challenges the understanding that negative symptoms always predict outcome (6). Finally, this study documents that is it possible to establish and maintain a strong alliance with this population and it is the first study to support the hypothesis that high attendance to treatment is associated with a therapeutic alliance as seen from the patient perspective.

### 4.1. Implications for practice

The findings in this study indicate that MT is a way to enhance the patient’s ability to form relation whereas ML does not have the same relational quality. Nevertheless, the patients that did complete the treatment had a measurable change in their level of negative symptoms, and the effect sizes in ML group were higher than in the MT group for some subscales. Additionally, the change in PANSS total was the same for both groups (see Table 3). If findings are confirmed in a new study with sufficient statistical power, it suggests that MT intervention can decrease negative symptoms and enhance quality of life and the ability to form an alliance with the music therapist. The ML interventions were also able to show reduction in negative symptoms but did not change QOL or the alliance.

### 4.2. Implications for research

The small size of the study makes conclusions difficult, as a larger sample size is needed to give the study the necessary power. An independent replication of the findings with a larger sample will be needed to strengthen our confidence in the findings. It would also be recommended that a treatment fidelity instrument would be included to ensure that the providers comply with the manual.

Interview data from participants could also broaden our understanding of the participants perspective on receiving the two interventions. Such data are also available and will be presented in a separate publication.

## 5. Conclusion

The analysis could not detect an association between Helping alliance and outcome variables as participants randomized to MT developed a strong alliance which was not observed in participants randomized to ML. No difference was observed on change in negative symptoms. This finding is surprising as working alliance in treatment is generally seen as an important factor for improvement. This finding differs from the general view on alliance in treatment. Caution is needed due to the low power of the study. The findings also documented a strong alliance development, lower dropout and higher attendance in treatment in MT group than in the ML group. The findings could indicate that MT is preferable over ML for prevention of dropout and increase of adherence to treatment.

## Data availability statement

The original contributions presented in the study are publicly available. This data and code can be found at: https://osf.io/huprd/.

## Ethics statement

The studies involving human participants were reviewed and approved by Den Videnskabsetiske Komité for Region Nordjylland. The patients/participants provided their written informed consent to participate in this study.

## Author contributions

IP, NH, and LB: conceptualization and investigation. IP and LB: resources and data curation. NH and CG: writing—original draft preparation. NH, CG, RN, LB and IP: writing—review and editing. IP: project administration and funding acquisition. All authors contributed to the article and approved the submitted version.

## Funding

This study owes thanks to the TRYG Foundation (ID110977) and the Obel Family Foundation for the funding of the study. We would also like to acknowledge Aalborg University, Department of Communication and Psychology and Aalborg University Hospital, Department of Psychiatry for joint financial support in the original study.

## Conflict of interest

The authors declare that the research was conducted in the absence of any commercial or financial relationships that could be construed as a potential conflict of interest.

## Publisher’s note

All claims expressed in this article are solely those of the authors and do not necessarily represent those of their affiliated organizations, or those of the publisher, the editors and the reviewers. Any product that may be evaluated in this article, or claim that may be made by its manufacturer, is not guaranteed or endorsed by the publisher.
