# Supplementary material for: Process-outcome relations in music therapy versus music listening for people with schizophrenia viewed through a mediational model: the role of the therapeutic alliance
Source: Front Psychiatry. 2023 May 2;14:1120003. doi: 10.3389/fpsyt.2023.1120003 (PMC10185766; doi:10.3389/fpsyt.2023.1120003)
Supplement: Supplementary file 1 [file data_sheet_1.pdf]

Supplementary Material

Appendix I. Summary of manuals used for the interventions in the study

Manual for the music therapy intervention (MT)

The manual initially described tree basic treatment principles. These principles are focused on the attitude and focus of attention of the therapist during treatment. They are as follows: Disciplined subjective, use of the relation, and timing.

The disciplined subjectivity principle instructed the therapist to be non-judgmental, accepting, and containing the participant constantly even if the patient was withdrawn or otherwise distant and passive. In relation to the musical expression or music-based experience it was essential not to impose interpretation, meaning or other therapist focused understandings. Beside this the music therapist should engage and motivate the participants. Using the relationship was related to using music in any way possible to engage and interact with the patients. Timing was related to when and how the therapist navigated in the therapeutic process. Often the therapist was following and mirroring the patients through the music in the first phase to facilitate connection and a beginning sense of being together. Later when suitable more differentiated positions in the musical relationship would be presented. These basic principles are outlined in the following four layers of the manual.

It was **unique and essential** that the therapist was able to navigate in and out of the participants mental state of mind. Negative symptoms should be viewed as something "between us", not as only belonging to the patient. The therapist should continuously motivate the participant to engage and participate in musical activities without using pressure or persuasion. The therapist should be mirroring and acknowledging to even small changes in the participants way of engaging. Subtle changes in eye contact, tone of voice, initiatives and content in verbal and/or expression.

It was **essential but not unique** that the therapist was attentive, curious, and focused on investigating different ways of understanding the participants perspective, and on repairing potential misunderstandings in the communication. The therapist should be able to self-regulate and attune responses. The therapist should offer a shared space to the participant where the level and the character of contact could vary as a means to prolong the time spend together. The therapist had to be aware of the 25-session time frame and prepare termination accordingly.

It was **acceptable** to offer advice or explanation when appropriate, to not use music in every session, and to give musical performance in the time frame of the planed session.

It was **not acceptable and prohibited** under any circumstances to pressure the patient to participate in anything against the person's will. The therapist was not allowed to use interventions governed by theoretical or methodological principals alone. The therapist should not be judgmental or dismissive towards the patient's experience and should always comply to the ethical standards for the music therapy profession.

There is no record of to what degree the therapist followed the manual, but the chief investigator was clinical supervisor for all therapist in both groups.

Manual for music listening intervention (ML)
This manual did not offer general instructions. It stated three levels: What must be done, what could be done, and what was not allowed.
What must be done: In the ML group the care person should be open, listening, and non-directive towards the content of the session. It was allowed to motivate and focus on music listening using the predesigned playlists (MusicStar app. Lund et al, 2016), whenever possible during the sessions. It was allowed to offer choices in music by playing short presentations of different music sources to support the patient's musical choice.
What could be done: The care person could choose to play a game, watch video together, draw or offer other activities beside music listening, but not as a replacement for music listening. A break during session was also possible.
What was not allowed: The care person was not allowed to actively inquire into the patient's actual life-situation or history and was not allowed to offer advice of any kind. If the patients initiate conversation about personal events, the care person could engage by listening in a non-exploring and non-confronting manner. The care person was not allowed to choose music unless asked by the patient. And if this happened, only music from the prepared playlist could be used. It was not allowed for the care person too share personal material; to actively engage musically with the patient; to bring their own music or to dialogue with the patient about how music listening influenced the patient.

Appendix II: Linear and generalised linear models

Model/predictor	Estimate (SE)	p-value
1.a) Outcome explained by intervention and moderators		
Negative symptoms (PANSS negative)		
Group	-0.4 (4.87)	0.936
Age	0.09 (0.31)	0.769
Duration of illness	-0.23 (0.45)	0.622
Gender	6.89 (3.93)	0.11
Quality of life (WHOQOL)		
Group	-8.67 (10.15)	0.415
Age	0.15 (0.62)	0.817
Duration of illness	0.2 (0.89)	0.826
Gender	-12.31 (7.89)	0.153
Functioning (GAF)		
Group	-4.59 (5.8)	0.447
Age	0.06 (0.37)	0.865
Duration of illness	-0.4 (0.54)	0.468
Gender	-5.14 (4.68)	0.298
Dropout before 25 weeks		
Group	2.65 (1.01)	0.009**
Age	-0.01 (0.06)	0.803
Duration of illness	0.05 (0.09)	0.601
Gender	1.36 (0.99)	0.169
1.b) Outcome explained by intervention x moderators		
Negative symptoms (PANSS negative)		
Group	-18.75 (19.37)	0.365
Age	-0.25 (0.48)	0.615
Duration of illness	0.08 (0.61)	0.899
Gender	2.14 (4.96)	0.679
Group x age	0.3 (0.66)	0.665
Group x duration of illness	0.62 (1.28)	0.642
Group x gender	12.51 (8.73)	0.195
Quality of life (WHOQOL)		
Group	-44.58 (43.91)	0.349
Age	-0.3 (1.04)	0.786
Duration of illness	0.51 (1.32)	0.711
Gender	-11.96 (10.67)	0.305
Group x age	0.87 (1.42)	0.561
Group x duration of illness	1.32 (2.78)	0.652
Group x gender	-3.12 (19.55)	0.879
Functioning (GAF)		
Group	-25.6 (23.68)	0.316

Age	-0.49 (0.59)	0.432
Duration of illness	0.33 (0.75)	0.678
Gender	-7.96 (6.07)	0.231
Group x age	0.95 (0.8)	0.275
Group x duration of illness	-2.42 (1.57)	0.168
Group x gender	2.32 (10.67)	0.834
Dropout before 25 weeks		
Group	9.24 (5.49)	0.093
Age	0.21 (0.15)	0.165
Duration of illness	-0.28 (0.2)	0.176
Gender	0.46 (1.56)	0.769
Group x age	-0.37 (0.19)	0.056
Group x duration of illness	0.77 (0.45)	0.088
Group x gender	4.25 (3.46)	0.219
2.a) Mediator explained by intervention		
Alliance (week 5)		
Group	-0.22 (0.22)	0.32
Alliance (week 15)		
Group	-0.2 (0.23)	0.393
Alliance (week 25)		
Group	-0.68 (0.32)	0.042*
Sessions		
Group	-6.17 (2.24)	0.008**
2.b) Mediator explained by intervention x moderators		
Alliance (week 5)		
Group	-1.16 (1.33)	0.397
Age	-0.04 (0.04)	0.354
Duration of illness	0.08 (0.05)	0.133
Gender	-0.07 (0.44)	0.869
Group x age	0.07 (0.04)	0.145
Group x duration of illness	-0.09 (0.06)	0.132
Group x gender	-0.6 (0.62)	0.349
Alliance (week 15)		
Group	-0.38 (1.76)	0.832
Age	0.01 (0.05)	0.826
Duration of illness	-0.01 (0.06)	0.935
Gender	-0.36 (0.51)	0.504
Group x age	0.02 (0.06)	0.777
Group x duration of illness	-0.02 (0.07)	0.838
Group x gender	-0.12 (0.79)	0.878
Alliance (week 25)		
Group	-1.26 (2.85)	0.676
Age	0.03 (0.07)	0.716

Duration of illness	-0.01 (0.08)	0.875
Gender	0.07 (0.68)	0.922
Group x age	0.05 (0.09)	0.629
Group x duration of illness	-0.04 (0.1)	0.715
Group x gender	-1.53 (1.35)	0.308
Sessions		
Group	-35.92 (16.42)	0.039*
Age	-0.77 (0.48)	0.119
Duration of illness	0.82 (0.63)	0.206
Gender	-0.99 (5.63)	0.862
Group x age	0.96 (0.54)	0.087
Group x duration of illness	-0.6 (0.7)	0.401
Group x gender	-2.47 (7.29)	0.737
3) Outcome explained by mediators		
Negative symptoms (PANSS negative)		
Alliance (week 5)	-2.46 (2.97)	0.419
Alliance (week 15)	0.67 (4.14)	0.873
Alliance (week 25)	-2.41 (4.05)	0.559
Sessions	0.08 (0.76)	0.914
Quality of life (WHOQOL)		
Alliance (week 5)	3.8 (6.21)	0.549
Alliance (week 15)	1.24 (8.65)	0.888
Alliance (week 25)	6.31 (8.48)	0.467
Sessions	1.96 (1.68)	0.261
Functioning (GAF)		
Alliance (week 5)	-6.77 (4.05)	0.112
Alliance (week 15)	9.15 (5.65)	0.123
Alliance (week 25)	3.2 (5.53)	0.57
Sessions	-1.92 (1.04)	0.081
Dropout before 25 weeks		
Alliance (week 5)	-0.67 (1.13)	0.556
Alliance (week 15)	1.4 (1.4)	0.318
Sessions	-0.45 (0.19)	0.016*

Note. Showing results of linear models (for continuous outcomes) and generalised linear models (for binary outcomes). Model intercepts not shown. Reference categories: gender: female; group: MT. Age and duration were measured in years. [1]Not possible for alliance at week 25.
